# Forming Process Prediction Model and Application of Laser Cladding for Remanufactured Screw Pump Rotors

**DOI:** 10.3390/ma18071673

**Published:** 2025-04-05

**Authors:** Haiying Zu, Yongpeng Liu, Sihui Chen, Xiang Jin, Weidong Ye, Mingyuan Sun, Zhongmin Xiao, Liming Yao

**Affiliations:** 1 School of Mechanical Science and Engineering, Northeast Petroleum University, Daqing 163318, China; zuhaiying@126.com (H.Z.); 19586151638@163.com (Y.L.); 18834364849@163.com (S.C.); 13653458637@163.com (X.J.); ywd75106@163.com (W.Y.); 2 School of Mechatronics Engineering, Harbin Institute of Technology, Harbin 150001, China; 2023111865@stu.hit.edu.cn; 3 School of Mechanical and Aerospace Engineering, Nanyang Technological University, 50 Nanyang Avenue, Singapore 639798, Singapore

**Keywords:** screw pump rotor, laser cladding remanufacturing, response surface method, forming process optimization, path planning

## Abstract

In order to achieve high-quality repair of complex curved parts, a remanufacturing process method utilizing laser cladding and reverse engineering technology is proposed to be implemented by robots. This study focuses on the oscillating helical surface of a screw pump rotor. A single-pass laser cladding test is conducted using Response Surface Methodology (RSM) to construct a predictive model and identify optimal process parameters. The model’s accuracy is validated through analysis of variance (ANOVA) and index verification, while the optimal lap rate is determined through multi-pass laser cladding testing. Using reverse engineering technology, the generation of laser cladding paths for complex surfaces is explored, and the trajectory planning for the laser cladding robot is carried out. Simulations and experiments of robotic laser cladding on complex surfaces are performed, with the optimal process parameters guiding both the experiment and simulation. The optimum single-pass cladding layer, with a lap rate of 25.6%, is achieved when the laser power is 2217 W, the powder feed rate is 2.86 r/min, and the scanning speed is 400 mm/min. The study successfully plans the path for laser cladding on complex curved parts, verifying its feasibility and effectiveness, verifying that there is good metallurgical bonding between the cladding layer and the substrate, and helping to select the appropriate process parameters that are consistent with the requirements of a particular application, thus providing valuable guidance for the remanufacture of failed metal parts.

## 1. Introduction

**Figure 1:**
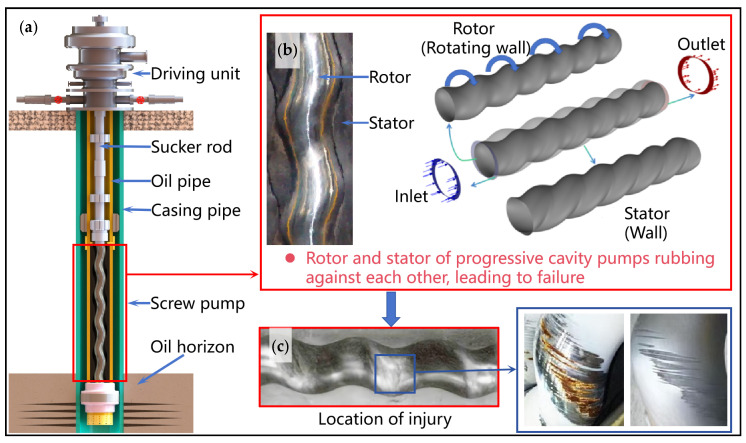
Screw rotor wear diagram: (**a**) Screw pump structure; (**b**) Screw pump rotor surface; (**c**) Rotor failure site.

Screw pumps (Figure 1a) are commonly used in the oil drilling industry for lifting high-viscosity heavy oil. The outer surface of the rotor in a screw pump is helically shaped to fit the inner surface of the stator, and the rotor meshes with the stator to form multiple sealed chambers (Figure 1b). Under the effect of the differential pressure at the pump’s intake, fluid or solid material is drawn in, and as the rotor rotates, the material is continuously pushed towards the discharge end, ultimately achieving the lifting of high-viscosity heavy oil [1]. The metal stator in an all-metal screw pump can withstand temperatures up to 400 °C, overcoming the temperature limitations of conventional rubber stators. During the transportation of high-temperature, high-pressure, high-viscosity solid/liquid two-phase (sand and oil) heavy oil, the meshing surfaces of the rotor and stator in the screw pump are highly susceptible to wear and failure (Figure 1c). In extreme cases, the pump may require overhauling or replacement as frequently as every 20 months [2]. Laser cladding, a surface enhancement technology, is widely employed in aerospace, automotive, and shipbuilding industries for component repair due to its low dilution rate, high density, strong metallurgical bonding, and fine-grained microstructure. Laser cladding for rotor repair offers minimal thermal deformation, high bonding strength, and dense cladding layers, which enhance rotor hardness, wear resistance, and corrosion resistance. Additionally, it substantially reduces repair costs [3].

In the process of remanufacturing the damaged surface of a rotor, the failure surface is irregular. When using laser cladding for repair and remanufacturing, the process starts with multi-pass surface repair from the edge of the failure surface, followed by surface-to-body repair using multi-layer deposition. The selection of laser cladding parameters and the planning of cladding paths have a direct impact on the effectiveness of the repair. Improper selection of parameters or improper planning of the cladding path can lead to defects such as residual stresses, bubbles, cracks, and other issues within the cladding layer [4]. Therefore, optimizing parameters and planning paths on the surface of the part to be repaired is a technical challenge that must be addressed. To address this issue, Huang Hanlin et al. [5] proposed a methodology for planning laser cladding paths for gears, based on region segmentation and trajectory refinement. This method uses a three-times NURBS surface reconstruction of the tooth surface model and extracts the failure region of the teeth through point cloud alignment and Boolean operations. Zu Haiying et al. [6] applied reverse engineering to acquire point cloud data of PDC drill bits and generated cladding models for the damaged areas. These data were then used for simulating and analyzing the repair trajectory in software, providing a reference for repairing complex surfaces using laser cladding remanufacturing technology. In a study by Wu yu et al. [7], the laser cladding process for 316L stainless steel was numerically simulated. The temperature and stress fields of the cladding layer were calculated under two scanning path scenarios, leading to the determination of the optimal scanning strategy. Yang et al. [8] employed Response Surface Methodology (RSM) and a Whale Optimization Algorithm–Bi-Directional Long Short-Term Memory (WOA-Bi-LSTM) model in laser-assisted additive manufacturing (LAAM) on Q235 steel. They utilized fitted equations derived from RSM and demonstrated that the multi-objective WOA-Bi-LSTM algorithm efficiently optimizes process parameters and enhances prediction accuracy. Zhang Wei-han et al. [9] proposed a surface TBC polishing path planning method for small turbine blades in aero-engines. By pre-processing the 3D point cloud of the blade and slicing it, polishing paths were generated using point cloud discretization and B-spline curve fitting, ensuring that the accuracy error stayed within 0.1 mm. Gao Senyao et al. [10] applied Taguchi’s orthogonal experimental design to investigate the effects of metal powder composition and processing parameters on laser-melted cladding coatings. They optimized the parameters using multi-response gray relational analysis (GRA). Validation experiments revealed a minimal deviation (0.95%) from predicted values, confirming the efficacy of GRA in optimizing laser cladding process parameters. Razavi R S et al. [11] designed an experiment using Response Surface Methodology to study the laser cladding of Inconel 718 on the same substrate under various laser process parameters. A prediction model for the morphology of the cladding layer was established, leading to the identification of the optimal combination of process parameters. Wang Pengfei et al. [12] conducted a combined experimental and simulation study to investigate the effects of process parameters and substrate tilt angle on the flow field and profile of the melting pool in GH3536. Their findings provide valuable guidance for adjusting process parameters and planning cladding paths. Gürkan K and Long H et al. [13,14] conducted Central Composite Design experiments to study the effects of key laser melting process parameters on the quality of the cladding layer, elucidating the mechanisms behind the enhanced wear and corrosion resistance of coatings. Chen et al. [15] fabricated AlCoCrFeNi alloy coatings on H13 steel via laser cladding. Using GRA, they analyzed the influence of process parameters on cladding quality and developed a multi-objective prediction model. This approach offers a framework for process analysis and optimization in other advanced manufacturing applications. Currently, for free path laser cladding repair of curved parts, traditional methods struggle to meet the required product quality standards. It is essential to combine processing technology with the changing curvature radius of the surface to develop shaping strategies that meet dimensional accuracy and improve processing flexibility—an urgent technical issue to resolve. Currently, research on laser cladding repair of screw pump rotors is limited. Using robots for laser cladding repair can reduce maintenance cycles and save costs. Therefore, research into robotic laser cladding repair of screw pump rotors is essential.

In order to improve the processing efficiency of laser cladding, precise control of the cladding layer morphology is essential. This paper presents a laser cladding experiment on the rotor of a failed screw pump. The response surface method is employed to design the experiment, investigating the effects of coupled laser cladding process parameters on the geometry and quality of the cladding layer. A prediction model is established to identify the optimal process parameter combination. Using reverse engineering, data acquisition and processing of the rotor surface are performed to obtain the solid model. The initial cladding path is determined by intersecting the truncated plane group with triangular surface patches. A robot-simulated surface laser cladding experiment is then conducted, providing a reference for repairing complex surface components using laser cladding technology.

## 2. Materials and Methods

### 2.1. Experimental Equipment and Materials

The objective of this study is to investigate an efficient and high-quality repair process for screw pump rotors. To this end, the cladding substrate used was 45 steel, the same material as the screw pump rotor. The dimensions of the substrate were 80 mm × 40 mm × 20 mm, and its chemical composition is listed in Table 1. The cladding powder’s composition and coefficient of thermal expansion were matched to those of the rotor to minimize residual stresses, reduce cracking, and ensure a strong metallurgical bond [16]. The cladding material was a mixed powder consisting of 80% 420 iron-based powder and 20% boron carbide powder. The selected 420 Fe-based powder exhibits high strength and wear resistance, with material properties similar to those of the rotor, ensuring strong matrix compatibility in the fusion cladding layer. Boron carbide, a super-hard ceramic material, exhibits high wear resistance. However, due to its inherent hardness and brittleness, it suffers from poor forming quality in the fusion cladding layer and cannot be used independently for coatings. Thus, it is employed as a hard reinforcing phase to enhance the hardness and wear resistance of the cladding layer [17,18], with a particle size range of 48–150 μm. The main chemical composition of the cladding material is shown in Table 2.

**Figure 2:**
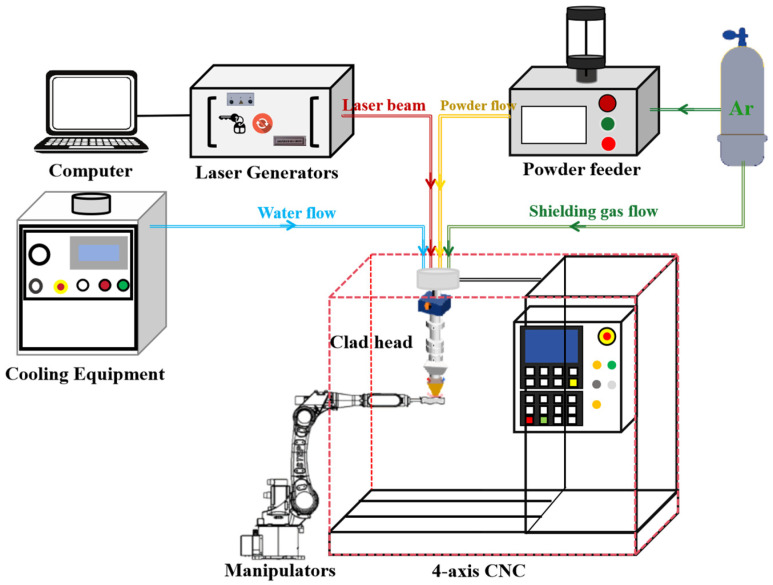
Laser cladding test system.

The laser cladding test system is shown in Figure 2. The system consists of an Excalibur-branded cooling unit, a fiber laser, a computer, a powder feeder, a cladding head, and a 4-axis CNC system. A SAMTLas 4S fiber laser with a wavelength of 1064 nm, a maximum power of up to 3000 W, and a spot diameter of 2.4 mm was used for the test. Prior to testing, the substrate surface was polished with sandpaper to remove rust and oxides, followed by cleaning with anhydrous ethanol to prevent oxidation. The protective gas used in the test was argon with a purity of 99.9% and a flow rate of 5 L/min. After the cladding process, the substrate was cut along the vertical weld direction to prepare metallographic specimens. The cross-section of the cladding layer was polished using sandpaper with grit sizes ranging from 150 to 1500, and the substrate surface was polished until no visible scratches remained. The specimens were then subjected to a 15 s corrosion treatment with 4% nitric acid in ethanol. The cross-sectional morphology of the cladding layer was analyzed using an OLYMPUS-GX71 metallographic microscope, and the morphological characteristics were measured.

### 2.2. Experimental Design and Results

#### 2.2.1. Single-Pass Laser Cladding Experiment

**Figure 3:**
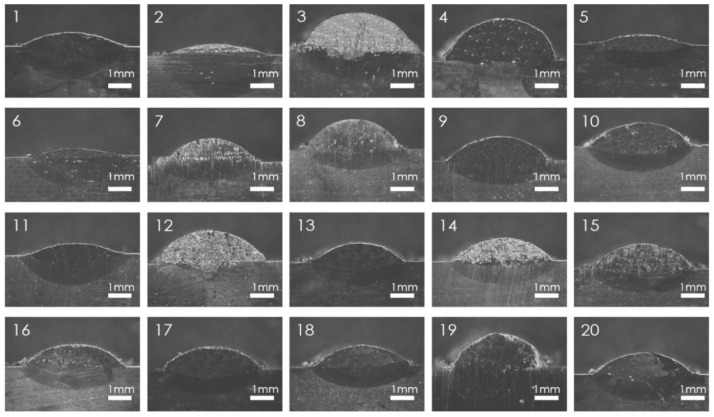
Specimen cladding section geometry topography.

In order to analyze the effects of different process parameters (laser power, powder feed rate, scanning speed) on the geometrical characteristics of the cladding layer (height, melt pool width, melt pool depth, and aspect ratio) and the quality of the cladding (dilution rate and microhardness), a 3-factor, 3-level experimental design was implemented using the Central Composite Design (CCD) module of Response Surface Methodology (RSM). The experiment involves a small specimen size and high accuracy of the expected results. The influencing factors in the laser cladding process were laser power (*P*), powder feed rate (*v_f_*), and scanning speed (*v_s_*). The laser power was set at 1.7, 2.0, and 2.3 kW, the powder feed rate at 1, 2, and 3 r/min, and the scanning speeds at 400, 600, and 800 mm/min. The factors were coded at levels of −1, 0, and 1, as shown in Table 3. The response variables selected to characterize the cladding layer morphology included the height (*h*), melt pool width (*w*), melt pool depth (*D*), and aspect ratio (*w*/*h*). For quality characterization, microhardness (HV) and dilution rate (*η*) were considered. A total of 20 experimental runs were designed based on these parameters, with the detailed scheme and results presented in Table 4. The morphology of the cladding layer is shown in Figure 3.

#### 2.2.2. Multi-Pass Laser Cladding Experiment

**Figure 4:**
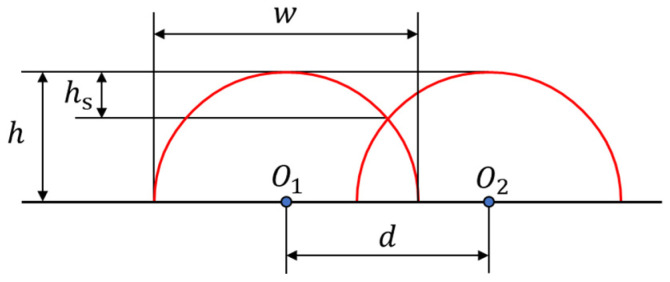
Schematic diagram of multi-channel overlapping laser cladding.

In order to achieve a uniform, smooth surface with excellent internal cladding quality during the cladding process, it is crucial to precisely control the overlap rate between individual cladding passes. The overlap rate is defined as the ratio of the lap width between adjacent passes to the width of a single pass in multi-pass cladding. The schematic of single-pass overlap in laser cladding is shown in Figure 4. A multi-pass overlap test was designed, where the center distance (*d*) and overlap rate (*K*) were calculated using Equations (1) and (2) based on the optimized process parameters for single-pass laser cladding. A total of 10 passes were selected for the lap test, and a single-factor experiment was conducted on the center distance (*d*) to determine the optimal overlap rate through experimental validation.

(1) $$d=\frac{{\left\{\frac{{\left(w/2\right)}^{2}+h^{2}}{2h}\right\}}^{2}\times \frac{\arcsin\left(w\times h\right)}{{\left(w/2\right)}^{2}+h^{2}}-\frac{\left(w/2\right)\times \left[{\left(w/2\right)}^{2}-h^{2}\right]}{2h}}{h}$$

(2) $$K=\frac{w-d}{w}$$

The parameters defining the cladding geometry are as follows: *h* represents the height of the single-pass cladding, *h*_s_ is the height difference between the crest and trough, *w* denotes the width of the single-pass cladding, and *d* is the center-to-center distance between adjacent single-pass claddings.

## 3. Results and Analysis

### 3.1. Geometrical Characterization of Cladding Layer

#### 3.1.1. Prediction Model for Cladding Layer Geometry

The objective is to construct a model that accurately reflects the relationship between the input variables and response values using limited experimental data. In this study, a regression prediction model was developed to estimate the response of input variables (laser power *P*, powder feed rate *v_f_*, and scanning speed *v_s_*) on response values (fused cladding layer height *h*, melt pool width *w*, melt pool depth *D*, and width-to-height ratio *w*/*h*). This model was constructed based on the experimental data presented in Table 4, as described in Equations (3)–(6). The resulting regression model was then used to analyze and predict the process parameters in order to identify the optimal parameter combinations, as shown in Table 5 and Table 6. Subsequently, the experimental data were subjected to analysis of variance (ANOVA) to examine the interaction effects of the process parameters.

(3) $$h=1.01-0.0851P+0.4424v_{f}-0.17v_{s}-0.1358Pv_{f}-0.0716Pv_{s}-0.1713v_{f}v_{s}$$

(4) $$\begin{array}{l} w=3.96-0.0354P+0.4768v_{f}-0.2101v_{s}+0.1056Pv_{f}+0.0409Pv_{s}\\ +0.0071v_{f}v_{s}+0.0321P^{2}-0.3098v_{f}^{2}-0.0834v_{s}^{2} \end{array}$$

(5) $$\begin{array}{l} D=0.7432+0.1397P-0.0352v_{f}-0.1039v_{s}+0.0056Pv_{f}\\ -0.0176v_{f}v_{s}-0.001P^{2}-0.1215v_{f}^{2}-0.0005v_{s}^{2} \end{array}$$

(6) $$w/h=4.1+0.0578P-1.12v_{f}+0.2003v_{s}+0.6097Pv_{f}+0.4771Pv_{s}+0.4927v_{f}v_{s}$$

In this formula, *P* represents the laser power, *v_f_* denotes the powder feed rate, and *v_s_* indicates the scanning speed.

Table 5 and Table 6 present the results of the analysis of variance (ANOVA) for the prediction models of cladding layer height (*h*), melt pool width (*w*), melt pool depth (*D*), and aspect ratio (*w*/*h*). The analysis of variance (ANOVA) assesses the significance of each factor by quantifying the proportion of variation in the response variable that is attributable to the variation in the independent variables, relative to the total variation. The *F-value* is a key indicator for evaluating model significance, with the *P-value* indicating the degree to which the *F-value* can be rejected. Generally, a larger *F-value* corresponds to a smaller *P-value*, indicating a more significant correlation. A *P-value* less than 0.05 is considered significant, less than 0.01 is highly significant, and less than 0.001 is extremely significant. This allows for a more intuitive assessment of the significance of each factor on the cladding layer’s geometry. The “Lack of Fit” term is used to assess the reliability of the fitted model, and the *R*^2^ value represents the correlation between the model and the experimental data. The closer *R*^2^ is to 1, the better the model’s correlation with the experiment. The signal-to-noise ratio evaluates the discrepancy between the measured and predicted values. If the ratio exceeds 4, it indicates a high level of confidence in the model, allowing for subsequent analysis.

As shown in the ANOVA table for the height of the cladding layer (*h*), melt pool width (*w*), melt pool depth (*D*), and aspect ratio (*w*/*h*), the *P-value* for the corresponding model coefficients is less than 0.0001, indicating that all four models are highly significant. In contrast, the *P-value* and *F-value* for the out-of-fit terms are both greater than 0.05, suggesting that these terms are non-significant and that the regression models fit well, indicating minimal experimental errors. The *R*^2^ values for the multivariate coefficients and the adjusted fit coefficient *R*^2^_Adj_ for all four models are close to 1, confirming excellent correlation for each model. The signal-to-noise ratios were 18.39, 18.90, 93.09, and 20.69, respectively, all exceeding 4, further supporting the high reliability of these models, which can be used to analyze and predict the parameters of the laser cladding process [19].

As shown in Table 5, the cladding layer height (h) and the melt pool width (w) are significantly influenced by the powder feeding rate and scanning speed (*P-value* < 0.0001). Laser power also plays a role, with a further comparison of F-values revealing that the process parameters affecting cladding layer height are ranked as follows: powder feeding rate > scanning speed > laser power. Similarly, the analysis of melt pool width indicates that the powder feeding rate and scanning speed have the greatest influence (*P-value* < 0.0001), followed by laser power. A further comparison of F-values reveals that the process parameters affecting melt pool width, in order of significance, are as follows: powder feeding rate > scanning speed > laser power. For the width-to-height ratio of the cladding layer and melt pool depth (Table 6), the analysis shows that laser power and scanning speed have the most significant impact on melt pool depth (*P-value* < 0.0001), while the powder feeding rate has the least effect. F-value comparisons further highlight the importance of these parameters, with the order of influence on melt pool depth being laser power > scanning speed > powder feeding rate. In terms of the cladding layer’s width-to-height ratio, the powder feeding rate is the most significant factor, followed by scanning speed and laser power. Overall, the three factors influence the geometrical characteristics of the cladding layer in the following order of significance: powder feeding rate > scanning speed > laser power.

**Figure 5:**
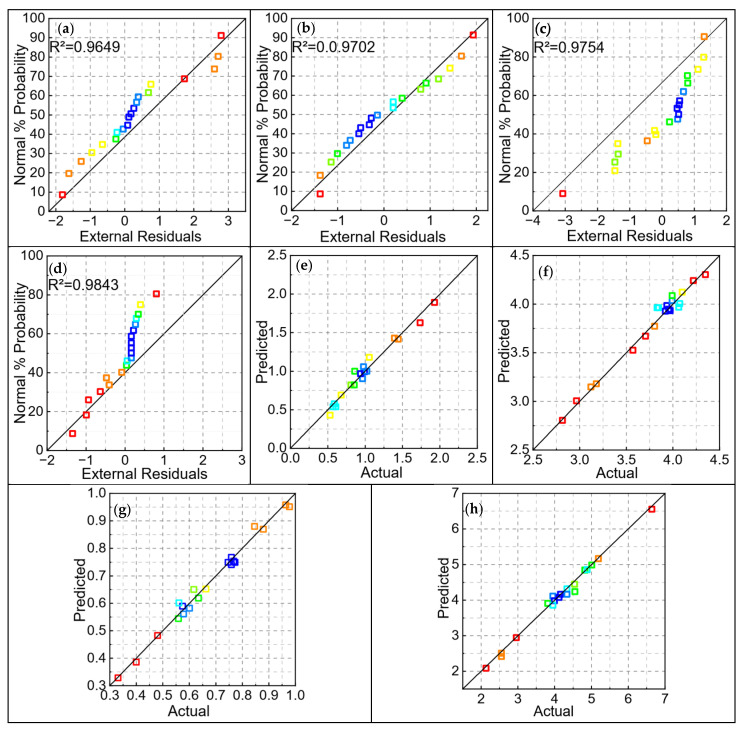
Residual normal distribution plots and comparison of predicted and experimental values for a model of geometric characteristics of fused cladding layers: (**a**) residuals of weld high; (**b**) residuals of weld width; (**c**) residuals of weld depth; (**d**) residuals of aspect ratio; (**e**) comparison of weld high; (**f**) comparison of weld width; (**g**) comparison of weld depth; (**h**) comparison of aspect ratio.

An evaluation of the four prediction models for the geometrical characteristics of the cladding layer is presented. Figure 5a–d show the normal distribution of the residuals for all four models. It can be observed that the residuals of the response variables are closely aligned with the reference line, suggesting that the models exhibit a high degree of reliability within a certain range. Figure 5e–h presents the comparison between the predicted values and the actual values for the four models. The majority of the data points from each test are distributed near the 45° line, indicating that the predicted values closely match the actual values. This demonstrates that the four models are capable of accurately predicting the response targets [20].

#### 3.1.2. Influence of Process Parameters on Cladding Layer Geometry

**Figure 6:**
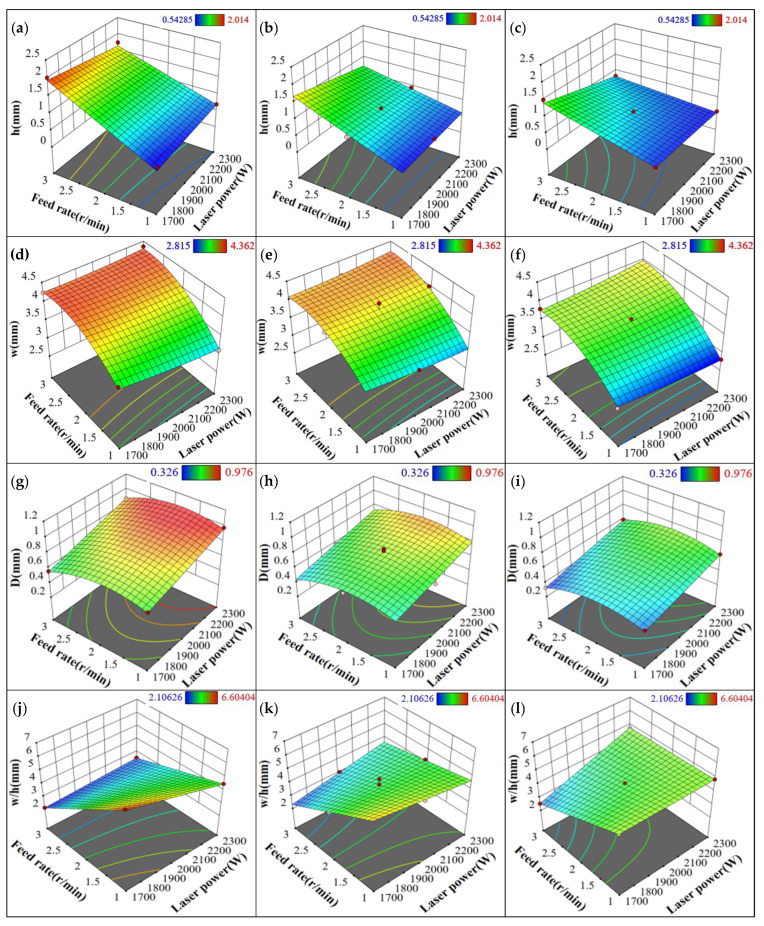
Response surfaces of height, width, depth, and aspect ratio of fused cladding layers at different scanning speeds: (**a**) 400 mm/min, height; (**b**) 600 mm/min, height; (**c**) 800 mm/min, height; (**d**) 400 mm/min, width; (**e**) 600 mm/min, width; (**f**) 800 mm/min, width; (**g**) 400 mm/min, depth; (**h**) 600 mm/min, depth; (**i**) 800 mm/min, depth; (**j**) 400 mm/min, aspect ratio; (**k**) 600 mm/min, aspect ratio; (**l**) 800 mm/min, aspect ratio.

**Figure 7:**
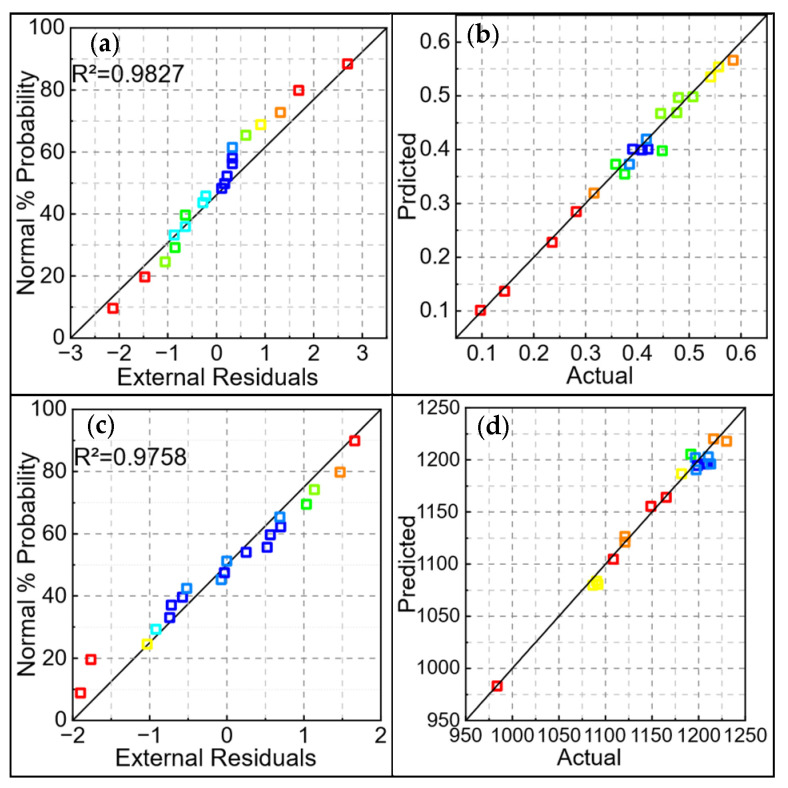
Plot of normal distribution of residuals and comparison of predicted and experimental values for model of quality of fused cladding layer: (**a**) residuals of dilution rate; (**b**) residuals of microhardness; (**c**) comparison of dilution rate; (**d**) comparison of microhardness.

Using the response regression prediction model, 3D response surface plots of the geometrical properties of the fused cladding layer under varying process parameters were obtained, as shown in Figure 6 and Figure 7 [21,22,23]. From Figure 6a–c, it is clear that at low scanning speeds, the 3D response surface has a steep slope, indicating that both the scanning speed and powder feeding rate significantly affect the height of the cladding layer. Increasing the powder feeding rate causes more powder to accumulate on the surface of the cladding layer, enhancing the melting of powder and significantly increasing the layer height. On the other hand, increasing the scanning speed reduces the laser beam’s contact time with the powder, decreasing energy input and preventing full melting, which in turn reduces the cladding height. Therefore, at constant laser power, a higher cladding height can be achieved by reducing the scanning speed and increasing the powder feeding rate. In Figure 6d–f, it is shown that at low scanning speeds, the slope of the 3D response surface is also steep. An increase in the powder feeding rate initially increases the melt pool width, but as the powder amount rises, the laser energy becomes insufficient for complete melting, causing the width to decrease. Additionally, increasing the scanning speed shortens the laser beam’s dwell time, reducing the energy absorbed by the substrate and decreasing both the melt pool size and cladding width. Therefore, at constant laser power, a balanced combination of powder feeding rate and scanning speed can effectively increase the melt pool width. From Figure 6g–i, it is evident that laser power is the primary factor affecting melt pool depth at low scanning speeds, with a steeper 3D response surface observed. As laser power decreases, the melt pool depth also decreases, as insufficient laser energy prevents complete melting of the powder. As scanning speed increases, the reduced laser beam dwell time results in lower energy absorption by the substrate, causing the melt pool depth to decrease. Therefore, increasing laser power and decreasing scanning speed can effectively increase the melt pool depth. Finally, as shown in Figure 6j–l, when the scanning speed is set at 800 mm/min, the width-to-height ratio of the cladding layer shows high sensitivity to changes in scanning speed and powder feeding rate, with the 3D response surface increasing substantially, reaching a maximum of 5. The energy absorbed by the cladding layer is inversely related to scanning speed—higher scanning speeds lead to lower energy input, reducing both the cladding height and width, though the height decreases more rapidly, increasing the width-to-height ratio. An increase in scanning speed reduces the energy delivered to the cladding layer, leading to a decrease in cladding height. However, the width-to-height ratio increases as scanning speed increases. Based on this analysis, to achieve a larger width-to-height ratio at the same scanning speed, the powder feeding rate can be adjusted to an optimal level, and scanning speed can be further increased.

The preceding analysis indicates the following effects of laser process parameters on the geometrical characteristics of the single-pass cladding layer: the powder feeding rate has the most significant impact on the cladding layer’s height, width, and aspect ratio, followed by scanning speed. Regarding melt pool depth, laser power plays a dominant role. A larger weld cross-sectional area correlates with better mechanical properties. Considering the combined influence of these factors on the geometrical characteristics of the cladding layer, the process parameters for laser cladding should prioritize higher laser power, a moderate powder feeding rate, and a lower scanning speed.

### 3.2. Cladding Layer Quality

#### 3.2.1. Cladding Layer Quality Prediction Model

The quality of the cladding layer is typically characterized by the dilution rate (*η*) and microhardness (HV) [24]. The dilution rate refers to the extent of compositional change in the cladding alloy caused by the mixing of the melting substrate during the laser cladding process. It is expressed as the percentage of the substrate alloy in the total cladding layer (*η*). The dilution rate is a critical factor in controlling the laser cladding process. An excessively high dilution rate can result in significant dilution of the cladding layer by the substrate, which may compromise the inherent properties of the cladding and increase the likelihood of cracking and deformation. On the other hand, a very low dilution rate may prevent the formation of a strong metallurgical bond between the cladding layer and the substrate, making the cladding layer more prone to spalling. Therefore, controlling the dilution rate is essential for achieving a high-quality cladding layer. By performing regression fitting on the experimental parameters and results in Table 4, regression coefficients were obtained and used to construct two prediction models for the dilution rate and microhardness of the cladding layer, as shown in Equations (7) and (8).

(7) $$\begin{array}{l} \eta =0.4343+0.0611P-0.112v_{f}-0.0204v_{s}+0.0262Pv_{f}+0.0251Pv_{s}\\ +0.0313v_{f}v_{s}-0.0055P^{2}-0.0292v_{f}^{2}-0.0008v_{s}^{2} \end{array}$$

(8) $$\begin{array}{l} \mathrm{HV}=1196.13-31.79P+48.58v_{f}+38.54v_{s}+4.82Pv_{f}+14.44Pv_{s}\\ +4.91v_{f}v_{s}-8.93P^{2}-39.79v_{f}^{2}-30.76v_{s}^{2} \end{array}$$

To assess the reliability of the fusion cladding quality prediction models, an ANOVA was performed on both models. As shown in Table 7, the *P-values* of both models are less than 0.0001, while the corresponding *P-values* and *F-values* for the misfit terms are both greater than 0.05, indicating that the misfit terms are not significant. This suggests that both models have high predictive accuracy. The *R*^2^ and adjusted *R*^2^_adj_ values for both models are close to 1, indicating strong correlation. The signal-to-noise ratios for the dilution ratio (5.56) and microhardness (43.36) are both greater than 4, demonstrating that both models have high reliability and are suitable for subsequent analyses.

As shown in Table 7, the ANOVA reveals that laser power and powder feeding rate have the greatest impact on the dilution rate of the fused cladding layer (*P-value* < 0.0001), followed by scanning speed. Further comparison of the *F-values* shows that the factors influencing the dilution rate are ranked as follows: powder feeding rate > laser power > scanning speed. Regarding the effect on the microhardness of the fused cladding layer, the scanning speed and powder feeding rate have the most significant impact (*P-value* < 0.0001), with laser power having a lesser effect. A further comparison of the *F-values* indicates that the factors influencing microhardness are ranked as follows: powder feeding rate > scanning speed > laser power.

An evaluation was conducted on the two models for predicting the quality of the fused cladding, as shown in Figure 7. This figure includes plots of the residuals’ normal distribution and a comparison between the predicted and actual values. From Figure 7a,b, it can be seen that the residuals for all response targets are generally concentrated around the straight line, indicating that the model is highly stable [25] and exhibits high reliability within the specified range, thus meeting the requirements for predicting the response targets. Additionally, as shown in Figure 7c,d, the data points from each test are evenly distributed around the 45° line, further demonstrating the high accuracy of the prediction model.

#### 3.2.2. Influence of Process Parameters on Cladding Layer Quality

**Figure 8:**
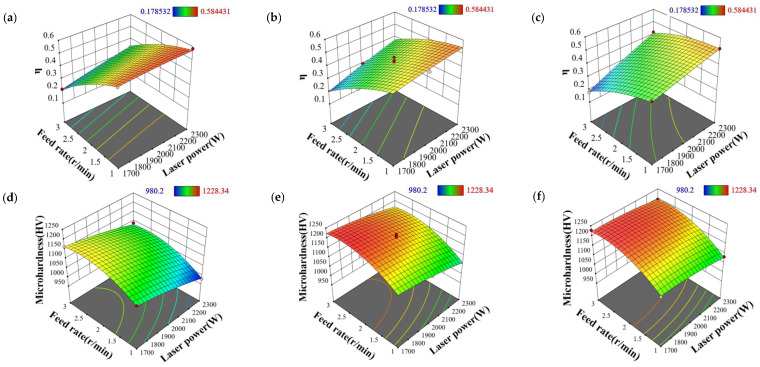
Response surfaces of dilution rate and microhardness of fused cladding layers at different scanning speeds: (**a**) 400 mm/min, dilution rate; (**b**) 600 mm/min, dilution rate; (**c**) 800 mm/min, dilution rate; (**d**) 400 mm/min, microhardness; (**e**) 600 mm/min, microhardness; (**f**) 800 mm/min, microhardness.

The cladding layer quality prediction model was used to generate 3D response surfaces for quality analysis under varying process parameters (Figure 8) [26]. As shown in Figure 8a–c, at high scanning speeds, the laser power and powder feeding rate have a significant impact on the dilution rate of the cladding layer, with the 3D response surface exhibiting a steeper slope. Increasing the powder feeding rate reduces the heat on the substrate and decreases the dilution rate of the cladding layer. Conversely, increasing the laser power raises the laser beam energy, which in turn increases the dilution rate of the cladding layer. It is also worth noting that the scanning speed has a minimal effect on the dilution rate. Therefore, at a constant scanning speed, reducing the powder feeding rate while increasing laser power can effectively enhance the dilution rate of the cladding layer. As shown in Figure 8e,f, at low scanning speeds, the laser power and powder feeding rate have a more pronounced influence on the microhardness of the cladding layer, leading to significant changes in the slope of the 3D response surface. An increase in the powder feeding rate tends to improve the microhardness of the cladding layer due to the large amount of powder being melted and adhering to the substrate, which strengthens the substrate surface and increases its microhardness. On the other hand, an increase in scanning speed also results in a rise in microhardness. The influence of laser power on microhardness is relatively small. Therefore, at constant laser power, increasing both the powder feeding rate and scanning speed can effectively enhance the microhardness of the cladding layer [27].

The preceding analysis reveals that the quality of the single-pass cladding layer is influenced by the laser process parameters in specific ways, with the powder feeding rate being the most critical factor. Considering the combined effects of all parameters on cladding layer quality, it is recommended to use moderate laser power, an appropriate powder feeding rate, and a lower scanning speed for optimal laser cladding.

## 4. Validation of Cladding Process Prediction Model

### 4.1. Experimental Verification of Single-Pass Laser Cladding

The primary aim of the model is to achieve precise control over the morphology of the cladding layer. To achieve this, the laser process parameters must be carefully adjusted to produce a cladding layer of optimal quality [28]. A moderate aspect ratio leads to well-formed cladding geometry, while a higher microhardness results in better cladding quality. The next step is to optimize the aspect ratio and microhardness of the cladding layer to validate the accuracy of the prediction model. The optimization conditions and objectives are outlined in Table 8.

**Figure 9:**
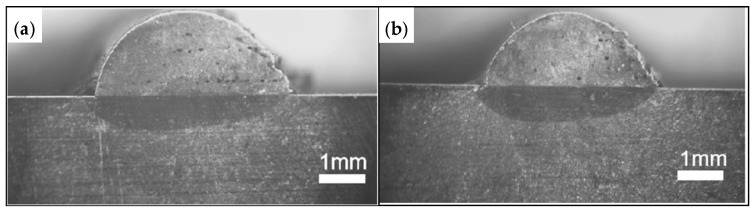
Optimization of test cladding cross-section: (**a**) Fusion 1; (**b**) Fusion 2.

The prediction model was used to determine the optimal combination of process parameters through comprehensive selection via software. The optimal parameters are a laser power of 2217 W, a powder feeding rate of 2.86 r/min, and a scanning speed of 400 mm/min. The results of the laser cladding experiment and the cross-sectional morphology are presented in Table 9 and Figure 9. As shown in Table 9, the discrepancy between the predicted and actual values for the aspect ratio of the cladding layer after optimization is only 1.9%. The error in the dilution rate is 4.8%, the error in microhardness is 1.2%, and the average error for all three parameters is a minimal 2.6%. These results demonstrate that the prediction model is highly accurate, capable of optimizing process parameters, and ensuring precise control over the morphology and quality of the fused cladding layer. The multivariate linear regression model developed by Shi et al. [29] achieved an overall average error of 5.19% and a maximum error of 7.14%, while models by Lian et al. [30] exhibited errors below 8% (maximum 7.89%), as shown in Table 10. These results demonstrate that the prediction model exhibits high accuracy and reliability, optimizes process parameters, and plays a critical role in precisely controlling cladding layer morphology/quality while improving cladding efficiency.

### 4.2. Experimental Verification of Multi-Pass Laser Cladding

Based on the optimal process parameters determined in Section 3.1, the height of the single optimal cladding layer *h* was found to be 1.066 mm, and its width *w* was 2.689 mm. By substituting these values into Equations (1) and (2), the theoretical spacing *d* between adjacent cut-off planes was calculated to be 2.001 mm, with an overlap ratio *K* of 25.6%.

**Figure 10:**
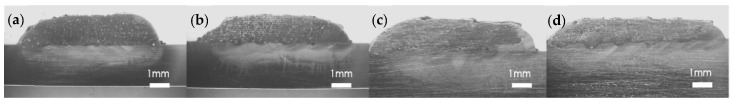
Cross-section of fused cladding at different lap rates: (**a**) 1.6 mm; (**b**) 1.8 mm; (**c**) 2.0 mm; (**d**) 2.2 mm.

The theoretical spacing between adjacent cut-off planes *d* was considered in the design of the optimal lap rate test, which involved 10 transverse laps. The center distance between adjacent laps was varied to determine the optimal value. To ensure the accuracy of the final result, the total width of the fused cladding and the total width of the lap were measured separately. The actual lap rate was then expressed as the ratio of the total width of the 10 laps to the total width of the fused cladding. The test results are presented in Table 11 and Figure 10.

When the center distance is 1.6 mm, the overlap rate is 40.5%. It can be observed that as the number of passes increases, the fused cladding layer shows a piling-up effect, with a tendency to gradually increase, which fails to meet the cladding requirements. When the center distance is 2.0 mm, the overlap rate is 25.6%. Examination of the cross-sectional morphology reveals that, except for the ends of the cladding layer, the middle part is essentially flat, and there is no significant bulging in the cladding layer. This presents the optimal morphology that meets the requirements. When the center distances are 1.8 mm and 2.2 mm, the topographic images show significant surface unevenness, with noticeable depressions between the two fused cladding layers, which do not meet the fusion cladding requirements.

### 4.3. Experimental Verification of Laser Cladding for 3D Repair

In view of the complex, curved surface of the screw pump rotor, surface normal vector irregularities pose significant challenges for laser cladding path planning. Path selection critically influences cladding accuracy, process efficiency, and ultimately governs the resultant microstructure and material properties. It is essential to carefully plan the laser cladding paths when repairing and reinforcing the damaged rotor surface.

**Figure 11:**
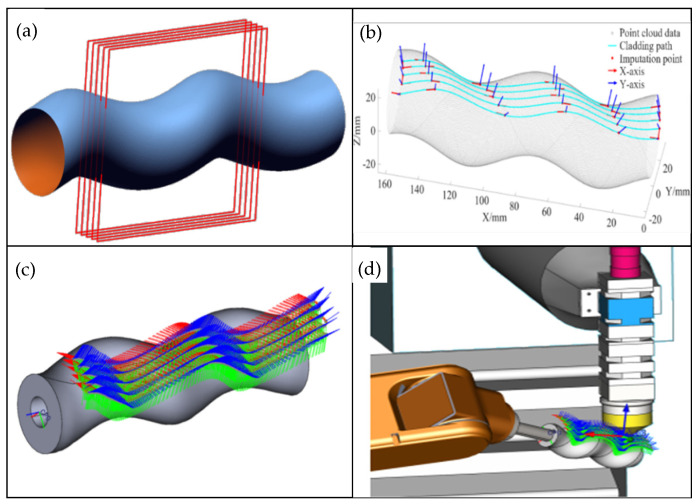
(**a**) Acquisition of laser cladding path; (**b**) interpolation point pose; (**c**) cladding simulation trajectory diagram; (**d**) robot attitude at trajectory point.

The screw pump rotor is modeled using reverse engineering, and the initial cladding path is derived through the intersection method of equidistant parallel planes and triangular surface patches [32], as shown in Figure 11a. The path is then discretized using the equal bow height method. To ensure the quality of the cladding, the direction of the laser beam must align with the normal vector of the interpolation points. Additionally, the K-neighborhood local surface fitting method is employed to estimate the normal vector. A Cartesian coordinate system is established according to the scanning sequence, with the transformed coordinate axis directions defined as unit vectors. The interpolated point positions are spliced together to form the interpolated point position matrix [33], as illustrated in Figure 11b. Kinematic analysis and trajectory planning are performed using the Xinshida SA1400 six-axis industrial robot, with the Cartesian coordinates of each joint calculated based on the joint coordinate system. The laser cladding path is smoothed by inserting transition arcs to ensure a smooth transition between path points [34]. The SA1400 robot is invoked within the PQart software environment, where the screw pump rotor STEP file is imported and linked with the trajectory data and the helical model. The simulation platform is configured to establish the coupling relationships among the robot, laser head, and screw rotor, ensuring a safety distance is maintained, preventing damage to the laser, and the interpolation points are extended 15 mm along the Z-axis. Simulation results show that the path length is 875.21 mm, with no issues such as unreachable points, axis overrun, or singularity (see Figure 11c,d). The optimal laser cladding process parameters for complex surface repair are selected, and these parameters are listed in Table 12.

**Figure 12:**
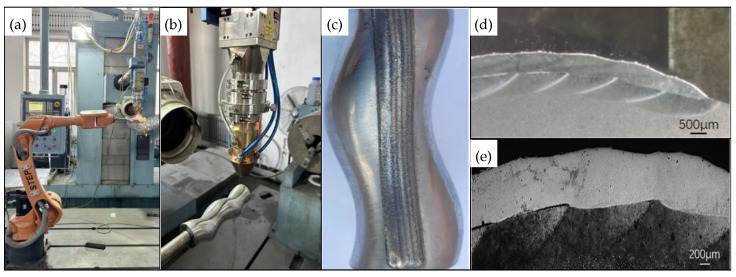
Experimental results of laser cladding: (**a**) manipulator; (**b**) laser cladding; (**c**) post-cladding; (**d**) cross-section of cladding layer 1; (**e**) cross-section of cladding layer 2.

In accordance with the final selected process parameters, the on-site experiment was conducted based on the path planning method outlined in the previous section. During the laser cladding process, the robot maintained the correct posture, avoiding axis overruns and singularity errors, and the operation was smooth and continuous [35]. The experimental results are shown in Figure 12. The longitudinal section of the cladded specimen corresponds to the case with a moderate overlap rate, where the thickness of the cladded layer is uniform, and its surface is nearly flat. The cladding quality is good, meeting the repair requirements. The overall surface of the cladded layer is smooth, with minimal waviness, and the forming quality is satisfactory. The simulation and verification of the laser cladding processing paths are conducted through offline programming technology. This approach provides a reference for the actual remanufacturing process, effectively reduces processing errors and debugging costs, and promotes its advancement toward digitization and intelligent automation.

### 4.4. Optimization of Microstructure and Performance Testing of Clad Specimens

#### 4.4.1. Microstructure of Optimized Cladding Specimen

**Figure 13:**
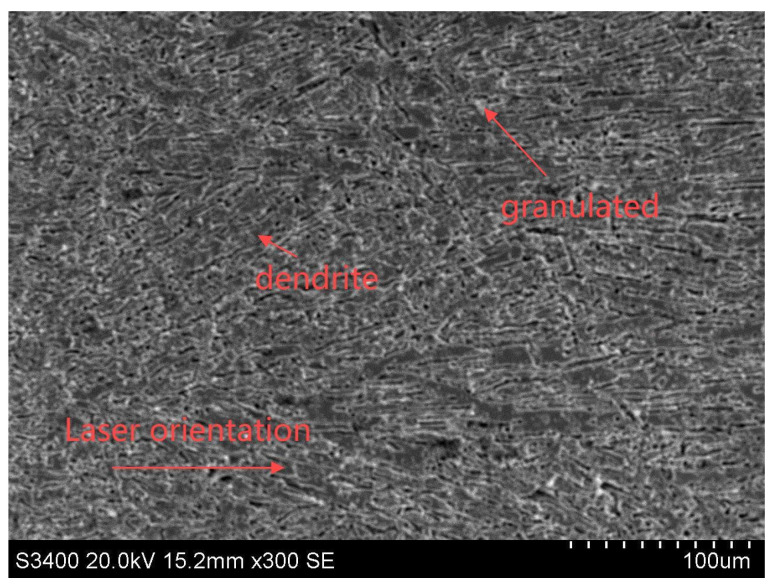
SEM images of coatings.

Following parameter optimization, the cladding’s microstructure and morphology were systematically characterized. Figure 13 presents the SEM micrographs of the clad layer, revealing distinct microstructural features. The coating exhibits martensitic dendrites aligned with the cladding direction, demonstrating epitaxial growth characteristics. The microstructure displays distinct layering with hierarchical dendrite development. Primary and secondary dendrites grow progressively, while boron carbide particles and intermetallics distribute interdendritically. These intermetallic phases appear as flake/needle structures between dendrites and matrix.

#### 4.4.2. Analysis of Coating Friction and Wear

**Figure 14:**
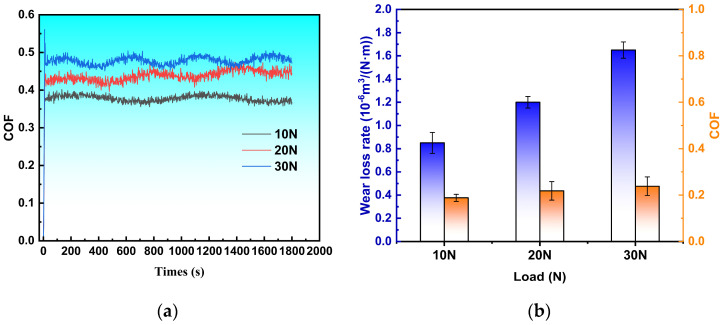
Friction and wear test results of fused cladding: (**a**) friction coefficient curve; (**b**) average friction coefficient and mass loss.

The friction coefficient curve characterizes the contact state of micro-motional wear in real time and serves as a key parameter for assessing wear kinetics [36]. To investigate the effect of fusion cladding on the wear resistance of 45 steel, friction tests were conducted on fusion-clad specimens using a tribometer. The results are shown in Figure 1. The curve evolution exhibits two characteristic stages: initial wear and stable wear. At the beginning of the test, the loaded counterface contacts the specimen surface, generating high shear stresses. The coefficient of friction increases rapidly during this stage. After 15 s, it stabilizes, and fluctuations diminish significantly. At a low load (10 N), the fused cladding exhibits the smoothest friction coefficient curve, with an average value of 0.376 and minor fluctuations, indicating stable wear conditions. When the load increases to 30 N, the mean friction coefficient rises to 0.476 (peak: 0.485), accompanied by significant fluctuations, indicating reduced wear stability. With increasing load (Figure 14b), the wear rate of the fused cladding rises significantly during the same test cycle, reaching mean values of 0.85, 1.2, and 1.65 at 10 N, 20 N, and 30 N, respectively. However, this increase was proportionally lower than the applied load increase. This may be attributed to crack formation and spalling caused by brittle fracture at higher loads, where spalled hard abrasive particles induce severe three-body abrasive wear. Notably, excessive boron carbide particles and intermetallics distribute interdendritically. These intermetallic phases appear as flake/needle structures between dendrites and matrix ceramic particle content triggers debonding, increasing the wear rate. Thus, moderate boron carbide addition enhances tribological properties [37].

## 5. Conclusions

(1) A prediction model for the morphology and quality of the cladding layer was developed using the Response Surface Methodology and experimental data, considering the interactions of process parameters. The optimal process parameter combination was found to be a laser power of 2217 W, a scanning speed of 400 mm/min, and a powder feeding rate of 2.86 r/min. Additionally, the overlap rate for multi-pass cladding was compared, with the optimal overlap rate determined to be 25.6%. The model’s validity was verified through single-pass to solid repair, demonstrating high accuracy. The results of multi-objective optimization can be used to optimize the combination of process parameters for specific application scenarios, providing a reference for optimizing and analyzing other advanced manufacturing technologies.

(2) The impact of process parameters on the geometry and quality of the cladding layer was analyzed through a combination of experiments and predictive models. The results show that the effects of each laser process parameter on the cladding layer morphology are as follows: the powder feeding rate has the most pronounced effect on the cladding layer height and melt pool width, followed by the scanning speed. Laser power was found to have the greatest influence on the melt pool depth, with scanning speed coming second. The aspect ratio was most significantly affected by the powder feeding rate, with scanning speed being a close second. Regarding the quality of the cladding layer, the powder feeding rate had the most significant impact on the dilution rate and microhardness, followed by laser power.

(3) Based on the optimization of process parameters, the surface laser cladding of a screw pump rotor was experimentally verified. The smooth surface and uniform thickness distribution of the fused cladding layer confirmed the effectiveness of the robot path planning strategy. This approach can be extended to the repair of compressor rotors, curved impeller blades, and similar components. In addition, the adaptive trajectory generation mechanism provides a reference for efficient free path programming of complex surfaces and aids in high-quality laser cladding remanufacturing of complex curved components.

## Figures and Tables

**Table 1:** Main chemical composition of screw rotor material.

				wt.%
C	Ni	Mn	Cr	Fe
0.42–0.5	≤0.25	0.5–0.8	≤0.25	Bal.

**Table 2:** Chemical composition of laser cladding 420 iron-based with boron carbide powder.

						wt.%
C	S	Mn	Cr	Si	B	Fe
0.35	0.03	0.29	0.12	0.1	0.18	Bal.

**Table 3:** Laser cladding process parameters and factor levels.

Level	*P* (W)	*v_f_* (r/min)	*v_s_* (mm/min)
−1	1700	1	400
0	2000	2	600
1	2300	3	800

**Table 4:** Design and results of cladding test based on response surface method.

Number	Factor			Response Value					
	*P* (W)	*v_f_* (r/min)	*v_s_* (mm/min)	*h* (mm)	*w* (mm)	*D* (mm)	*w*/*h*	*η* (%)	Microhardness (HV)
1	1700	1	400	0.543	3.585	0.66	6.604	54.945	1089.20
2	2300	1	400	0.694	3.123	0.98	4.500	58.443	980.20
3	1700	3	400	2.014	4.242	0.56	2.106	21.726	1162.72
4	2300	3	400	1.745	4.362	0.87	2.499	33.346	1088.99
5	1700	1	800	0.591	2.954	0.41	5.001	40.912	1119.18
6	2300	1	800	0.579	2.815	0.63	4.864	52.100	1083.93
7	1700	3	800	1.500	3.799	0.33	2.533	17.853	1028.34
8	2300	3	800	0.821	3.923	0.59	4.776	41.885	1196.34
9	1700	2	600	0.992	3.985	0.57	4.018	36.413	1213.37
10	2300	2	600	0.950	3.988	0.85	4.200	47.200	1145.45
11	2000	1	600	0.619	3.186	0.60	5.146	49.257	1107.60
12	2000	3	600	1.369	4.105	0.58	2.999	29.614	1189.50
13	2000	2	400	1.053	4.011	0.85	3.808	44.601	1118.24
14	2000	2	800	0.858	3.731	0.57	4.348	39.954	1196.92
15	2000	2	600	1.040	4.108	0.75	3.950	41.933	1205.30
16	2000	2	600	1.001	4.123	0.78	4.120	43.740	1174.50
17	2000	2	600	0.981	3.877	0.77	3.950	43.995	1203.20
18	2000	2	600	0.949	3.908	0.76	4.120	44.451	1209.59
19	2000	2	600	0.864	3.928	0.77	4.545	47.100	1199.80
20	2000	2	600	0.985	3.831	0.76	3.889	43.682	1215.60

**Table 5:** Analysis of variance table for height and width of cladding layer.

Source		Height of Cladding Layer					Width of Cladding Layer				
		Sum of Squares	*df*	Mean Square	*F-Value*	*P-Value*	Sum of Squares	*df*	Mean Square	*F-Value*	*P-Value*
**Model**		2.74	6	0.4570	59.55	<0.0001	3.43	9	0.3807	36.23	<0.0001
**Laser power**		0.0724	1	0.0724	9.43	0.0890	0.0125	1	0.0125	1.19	0.3004
**Powder feed rate**		1.96	1	1.9600	255.02	<0.0001	2.2700	1	2.2700	216.37	<0.0001
**Scanning speed**		0.2891	1	0.2891	37.66	<0.0001	0.4414	1	0.4414	42.01	<0.0001
**Residual**		0.0998	13	0.0077	-	-	0.1051	10	0.0105	-	-
**Lack of fit**		0.0819	8	0.0102	2.87	0.1303	0.0294	5	0.0059	0.3881	0.8389
**Pure error**		0.0178	5	0.0036	-	-	0.0757	5	0.0151	-	-
***R* ^2^**		0.9649		*R* ^2^_Adj_	0.9487		0.9702		*R* ^2^_Adj_	0.9435	

-: Residual and Pure error do not show their *P-value* and F-value data when outputted by Design-Expert 13 software, while when analyzing the other data in the tables, it can be shown that the established prediction model has a good prediction accuracy.

**Table 6:** Analysis of variance table for depth and aspect ratio of cladding layer.

Source		Depth of Cladding Layer					Aspect Ratio of Cladding Layer				
		Sum of Squares	*df*	Mean Square	*F-Value*	*P-Value*	Sum of Squares	*df*	Mean Square	*F-Value*	*P-Value*
**Model**		0.4794	9	0.0533	44.07	<0.0001	19.72	6	3.29	136.24	<0.0001
**Laser power**		0.1952	1	0.1952	161.47	<0.0001	0.0335	1	0.0335	1.39	0.2601
**Powder feed rate**		0.0124	1	0.0124	10.25	0.095	12.55	1	12.55	520.13	<0.0001
**Scanning speed**		0.1932	1	0.1932	159.85	<0.0001	0.4014	1	0.4014	16.64	0.0013
**Residual**		0.0121	10	0.0012	-	-	0.3136	13	0.0241	-	-
**Lack of fit**		0.0116	5	0.0023	25.66	0.14	0.0256	8	0.0032	0.0556	0.9996
**Pure error**		0.0005	5	0.0001	-	-	0.2880	5	0.0576	-	-
***R* ^2^**		0.9754		*R* ^2^_Adj_	0.9533		0.9843		*R* ^2^_Adj_	0.9771	

-: Residual and Pure error do not show their *P*-value and F-value data when outputted by Design-Expert 13 software, while when analyzing the other data in the tables, it can be shown that the established prediction model has a good prediction accuracy.

**Table 7:** Analysis of variance table for fused cladding dilution and microhardness.

Source		Dilution Rate of Cladding Layer					Microhardness of Cladding Layer				
		Sum of Squares	*df*	Mean Square	*F-Value*	*P-Value*	Sum of Squares	*df*	Mean Square	*F-Value*	*P-Value*
**Model**		0.1892	9	0.021	63.2	<0.0001	74,763.57	9	8307.06	44.72	<0.0001
**Laser power**		0.0374	1	0.0374	112.35	<0.0001	10,106.04	1	10,106.04	54.40	0.494
**Powder feed rate**		0.1237	1	0.1237	372.04	<0.0001	23,598.22	1	23,598.22	127.04	<0.0001
**Scanning speed**		0.0041	1	0.0041	12.46	0.054	14,850.23	1	14,850.23	79.94	<0.0001
**Residual**		0.0033	10	0.0003	-	-	1857.58	10	185.76	-	-
**Lack of fit**		0.0019	5	0.0004	1.36	0.3734	844.27	5	168.85	0.8332	0.5769
**Pure error**		0.0014	5	0.0003	-	-	1013.31	5	202.66	-	-
***R* ^2^**		0.9827		*R* ^2^_Adj_	0.9672		0.9758		*R* ^2^_Adj_	0.9539	

-: Residual and Pure error do not show their *P-value* and F-value data when outputted by Design-Expert 13 software, while when analyzing the other data in the tables, it can be shown that the established prediction model has a good prediction accuracy.

**Table 8:** Optimization conditions and objectives.

Name	Objectives	Lower Value	Upper Value
***P* (W)**	realm	1700	2300
***v_f_* (r/min)**	realm	1	3
***v_s_* (mm/min)**	realm	400	800
***w*/*h***	realm	2.1	6.6
**Microhardness (HV)**	maximum values	980	1215

**Table 9:** Comparison of optimized test prediction and actual value.

	*P* (W)	*v_f_* (r/min)	*v_s_* (mm/min)	*w*/*h*	*η* (%)	Microhardness (HV)	Degree of Credibility
**Projected value**	2217	2.86	400	2.59	34.9	1100	0.928
**Test value**	2217	2.86	400	2.53	32.9	1084	-
	2217	2.86	400	2.55	33.5	1090.7	-
**Average error**	-	-	-	1.9%	4.8%	1.2%	-

**Table 10:** Error results of model validation.

Reference	Model	Powder	Error
[8]	WOA-Bi-LSTM	SS420	0.206% (maximum)
[10]	GRA	FeCuNIiCrAl	0.95 (maximum)
[29]	Regression analysis	340Fe	5.19% (average)
[30]	Regression analysis	W6Mo5Cr4V2	8% (maximum)
[31]	GRA	Ni60/WC	7% (average)

**Table 11:** Experimental results for lap ratio.

Number	*P* (W)	*v_f_* (r/min)	*v_s_* (mm/min)	*d* (mm)	Total Width (mm)	Theoretical Width (mm)	Overall Width of Lap (mm)	*K* (%)
1	2217	2.86	400	1.6	19.21	20.7	7.78	40.5%
2				1.8	20.17		6.68	33.1%
3				2.0	20.66		5.29	25.6%
4				2.2	21.12		3.84	18.2%

**Table 12:** Laser cladding process parameters for complex surfaces.

Parameter	*P* (W)	*v_f_* (r/min)	*v_s_* (mm/min)	*K* (%)	Defocusing Amount (mm)
Value	2217	2.86	400	25.6	15

## Data Availability

The research data has been reflected in the article, please contact the authors if needed.

## Abbreviations

The following abbreviations are used in this manuscript:

ANOVA	Analysis of variance
RSM	Response Surface Methodology
CCD	Central Composite Design
GRA	Gray Relationship Analysis
LAAM	Laser-assisted additive manufacturing
WOA-Bi-LSTM	Whale Optimization Algorithm–Bi-Directional Long Short-Term Memory
*P*	Laser power
*v_f_*	Powder feeding rate
*v_s_*	Scanning speed
*h*	Height of cladding
*w*	Width of cladding
*d*	Center distance
*h*_s_	Height difference
*D*	Depth of cladding
*η*	Dilution rate
